# Performance of novel constructed wetlands for treating solar septic tank effluent

**DOI:** 10.1016/j.scitotenv.2020.142447

**Published:** 2021-02-01

**Authors:** Thammarat Koottatep, Tatchai Pussayanavin, Sopida Khamyai, Chongrak Polprasert

**Affiliations:** a School of Environment, Resources and Development, Asian Institute of Technology, Thailand; b Faculty of Science, Ramkhamhaeng University, Thailand; c Thammasat School of Engineering, Thammasat University, Thailand

**Keywords:** Multi-soil layer based system, Modified constructed wetland, Post-treatment, Solar septic tank effluent, Long term performance

## Abstract

To improve treatment performance of the solar septic tank technology, novel constructed wetland systems have been proposed as an effective post-treatment system. This study aimed to investigate the treatment performance of the multi-soil layer based constructed wetland (MSL-CW) and comparing with the modified constructed wetland (mCW) for treating solar septic tank effluent in long-term operation. Pilot-scale MSL-CW and mCW units were operated in parallel under the same conditions during the period of 2016–2019. Removal efficiencies of TCOD, SCOD and TBOD in the MSL-CW were not significantly different (*p* < 0.05) from those of the mCW unit, which were 70–72%, 63–68% and 78–82%, respectively. The removal efficiencies of TSS, TKN, NH_4_-N and TP were found in the same magnitude in both units. The total coliform and E.coli counts in the effluent of MSL-CW and mCW units were reduced from 10^5^ MPN/100 mL to be lower than 10^3^ MPN/100 mL. These long-term operational results demonstrated that the effluent from the MSL-CW and mCW units could meet the global standards of non-sewered sanitation systems and the WHO guidelines. The effects of seasonal variations and plant harvesting on the monthly treatment performance are discussed in this study.

## Highlights

• The MSL-CW and modified CW systems are considered as novel constructed wetlands for treating solar septic tank effluent.
• The systems are very effective for removing organic matters, solids, nutrients and pathogens in long term.
• The effluent could meet the global standards of non-sewered sanitation systems and the WHO guidelines.

## Graphical abstract

**Figure:**
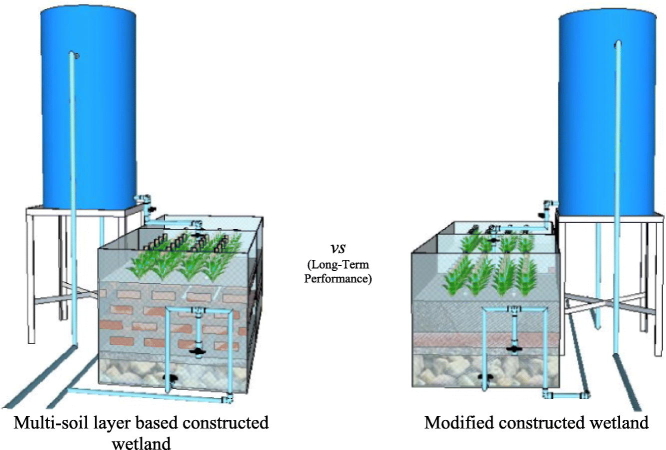

## 1 Introduction

The United Nations and World Health Organization (UNICEF and WHO, 2020) reported that more than 4.2 billion people worldwide still live without access to basic sanitation facilities and safely managed sanitation services, resulting in diarrheal infections and about 1 million deaths every year, especially children. Due to poor sanitation, human wastes such as feces, urine and cleansing water are discharged to the environment without suitable treatment which can cause environmental pollution and health risks to the people. To create a platform of global sanitation sustainability and overcome the remaining challenges toward the Sustainable Development Goal no. 6 (SDG6) on “Safely-Managed Sanitation”, the Bill & Melinda Gates Foundation, since 2011, has invested several hundreds million US$ to reinvent the sanitation technologies. One of the effective reinvented sanitation technologies to treat human wastes onsite is “Solar Septic Tank (SST)” which utilizes solar-heated water to increase temperatures inside the septic tanks, resulting effective biodegradation and pathogen inactivation. The solar septic tank prototypes have been investigated in several studies to verify their functionality, durability and performance (Pussayanavin et al., 2015; Polprasert et al., 2018; Connelly et al., 2019). As a new sanitation paradigm (Koottatep et al., 2019) at the global level to improve the region's environment and achieve the SDG6, the treated wastewater from the non-sewered sanitation systems should be designed to achieve both the national standards and the global standard (ISO30500). Thus, the existing treatment methods for the urban/rural context have to shift the design concept from “end-of-pipe” to “reuse, recycle, and resource recovery”. To improve performance of the solar septic tank technology in terms of treatment efficiencies, cost, and ease of maintenance, the novel constructed wetland systems have been proposed as effective post-treatment systems.

The novel constructed wetlands such as the multi-soil layer based constructed wetland (MSL-CW) and modified constructed wetland (mCW), at the laboratory-scale, were found to be able to remove more than 80% of both organic and nitrogen in the septic tank effluent (Koottatep et al., 2018). The MSL-CW consists of novel media that support the growth of heterogenous bacteria (for COD removal) and nitrifying-denitrifying bacteria (for NH_4_-N removal) and enhance the wastewater's infiltration ability to avoid clogging problems. The structure of MSL enables this CW system to be operated at shorter hydraulic retention time (HRT) and reducing area requirement (Koottatep et al., 2018; Masunaga et al., 2007; Ge et al., 2019; Von Sperling and de Lemos Chernicharo, 2005), applicable for urban areas of developing countries. The MSL-CW was previously studied for the removal of organic matter, nutrients and pathogens from various kinds of wastewater with satisfactory performance (Nguyen et al., 2019; Licciardello et al., 2018; Liang et al., 2020). However, there are few studies on the long-term performance or potential limitations of the MSL-CW in treating septic tank effluent. Thus, this study aimed to investigate the treatment performance of the MSL-CW and comparing with the modified constructed wetland (mCW) in treating solar septic tank effluent in long-term operation. The pilot-scale MSL-CW and mCW units were operated in parallel during the period of 2016–2019 under the same conditions (unit configurations, plant species and operational parameters).

## 2 Methodology

**Fig. 1:**
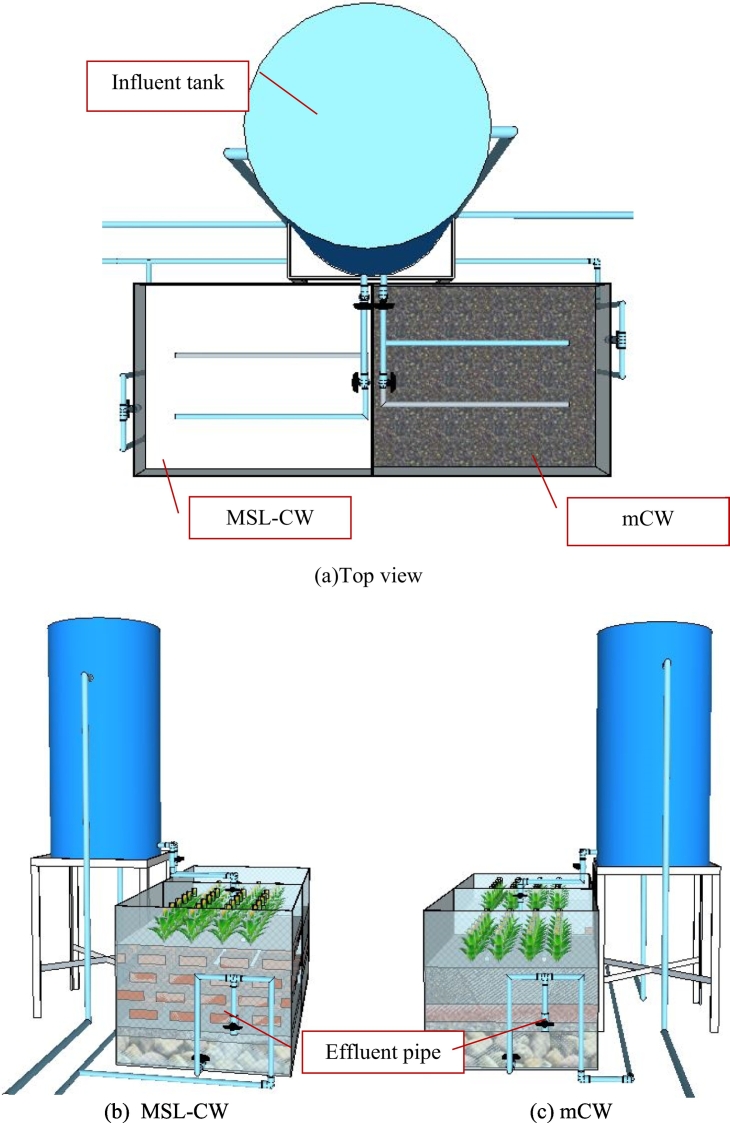

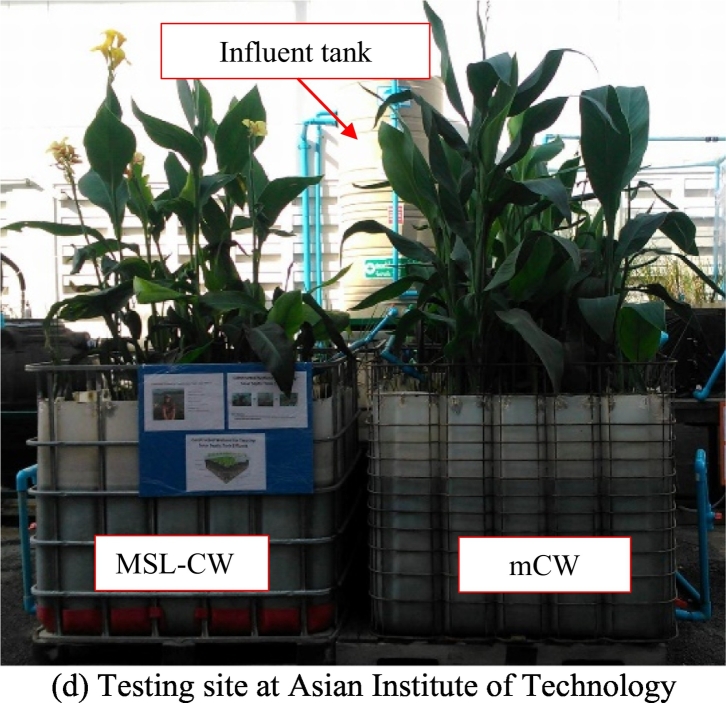
Experimental set up.

The pilot-scale MSL-CW and mCW units, made of rectangular plastic tanks, were installed and operated in parallel at the Asian Institute of Technology campus, Pathumthani, central Thailand in 2016 (Fig. 1). Dimensions of each experimental unit were 94 cm (width) × 115 cm (length) × 87 cm (height), with a surface area of 1.08 m^2^. The influent wastewater was evenly distributed by perforated pipes installed on the top of each unit (Fig. 1). Canna sp. was planted on the zeolite of the MSL-CW unit and on the clay loam layer of the mCW unit (see detail below section), and the space between each plant was about 15 cm. Plant harvesting was done 3 times a year or during the month of January, May and September. The influent wastewater used to feed the MSL-CW and mCW units was collected from the effluent of a solar septic tank (SST) unit operated at an average HRT of 24 h (Pussayanavin et al., 2020). The SST effluent with an average flow of 743 ± 375 L/day was then intermittently pumped to the influent tank (750 L in size), and subsequently overflowed to the MSL-CW and mCW units (Fig. 1), corresponding to organic loading rate of 54 ± 36 g TCOD/m^2^.d. All units were equipped with effluent pipes (or vertical tubes) to collect the effluent samples and control the water inside at a level of 60 cm from the bottom. Characteristics of the SST effluent in this study are depicted in Table 1. After the acclimatization stage, these two units, operated at ambient temperatures, were monitored in parallel from September 2016 to July 2019.

**Table 1:** Characteristics of the solar septic tank effluent (the average values during 2016–2019).

Parameters^a^^,^^b^	TCOD^c^ (mg/L)	SCOD^d^ (mg/L)	TBOD (mg/L)	TKN (mg/L)	NH^4+^-N (mg/L)	TP (mg/L)	TSS (mg/L)	Total coliform (10^5^ MPN/100 mL)	E.coli (10^5^ MPN/100 mL)
Mean	166	89	70	65	56	6	45	6.6	6.2
SD (±)	112	46	43	34	34	5	33	1.8	1.8

a Based on 80 numbers of samples during 2016–2019.

b The solar septic tank effluent distribute the flow equally (with the composition of 50/50 of total the volume) to each the MSL-CW and mCW units.

c Total chemical oxygen demand (TCOD).

d Soluble chemical oxygen demand (SCOD).

**Fig. 2:**
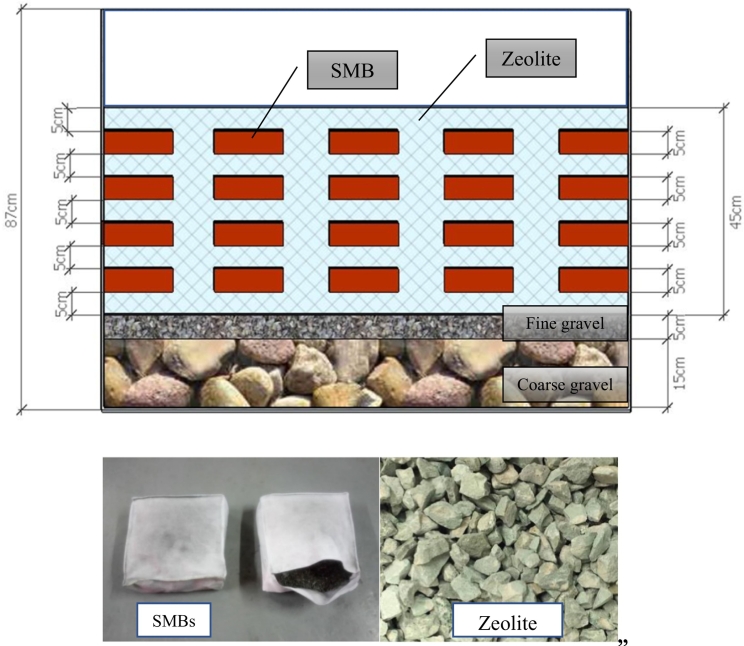
MSL-CW.

**Fig. 3:**
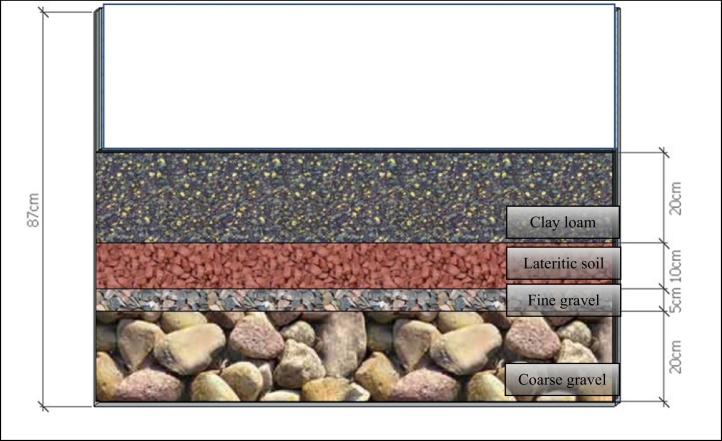
mCW.

The MSL-CW unit consisted of soil mixture block (SMBs), permeable layer (PLs) and underdrain layers (Fig. 2). The SMBs was the mixed composition on dry weight basis of lateritic (or iron-rich) soil (80%), sawdust (10%) and charcoal (10%). Powdered charcoal with sizing smaller than 0.1 mm was the porous material. The mixtures of SMBs were packed into fiber bags, each with a size of 15 cm (width), 5 cm (height) and 15 cm (length). The PLs comprised of zeolite (a clinoptilolite type) approximately 3–5 mm in diameter. The underdrain layers consisted of 15 cm of coarse gravel (3–5 cm in diameter) at the bottom, 5 cm of fine gravel (2–3 cm in diameter) in the middle (Fig. 2). The mCW unit consisted of four layers of 20 cm of clay loam (size smaller than 3 cm) at the top, 10 cm of lateritic soil (size smaller than 3 cm and Fe content of 20 g/kg) at the second layer, 5 cm of fine gravel at the third layer, and 20 cm of coarse gravel at the bottom (Fig. 3).

### 2.1 Sampling and chemical analyses

The influent (INF) and effluent (EFF) samples of the MSL-CW and mCW units were collected biweekly during the 2016–2017 and once a month during the 2018–2019 for analysis of total chemical oxygen demand (TCOD), soluble chemical oxygen demand (SCOD), total biochemical oxygen demand (TBOD), total suspended solids (TSS), total Kjeldahl nitrogen (TKN), ammonium (NH_4_-N), total phosphorus (TP), total coliform and E.coli, according to the standard methods (APHA, 2017).

### 2.2 Statistical analyses

Open-source R software was used for statistical analyses and graphics of the data. Statistical difference (at a 95% confidential level) of the relationship between the treatment units and its efficiencies was done by analysis of the variance (ANOVA), and comparing the multiple means was assessed using poc-host test (Tukey's Honest Significant Difference (HSD).The percent removal efficiency (re) was calculated according to Eq. (1).

(1) $$\text{Removal efficiency}\;\left(\%\right)=\left[\left(\text{Influent}-\text{Effluent}\right)/\text{Influent}\right]\;x\;100$$

## 3 Results and discussion

### 3.1 Treatment performance

**Fig. 4:**
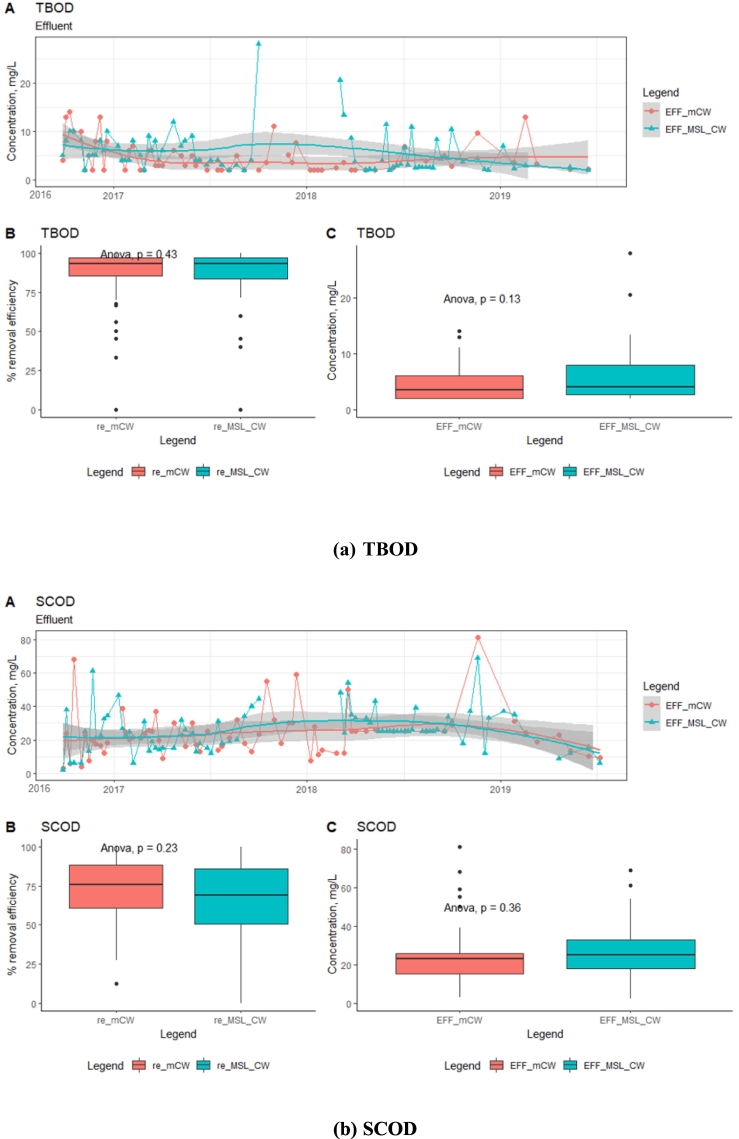

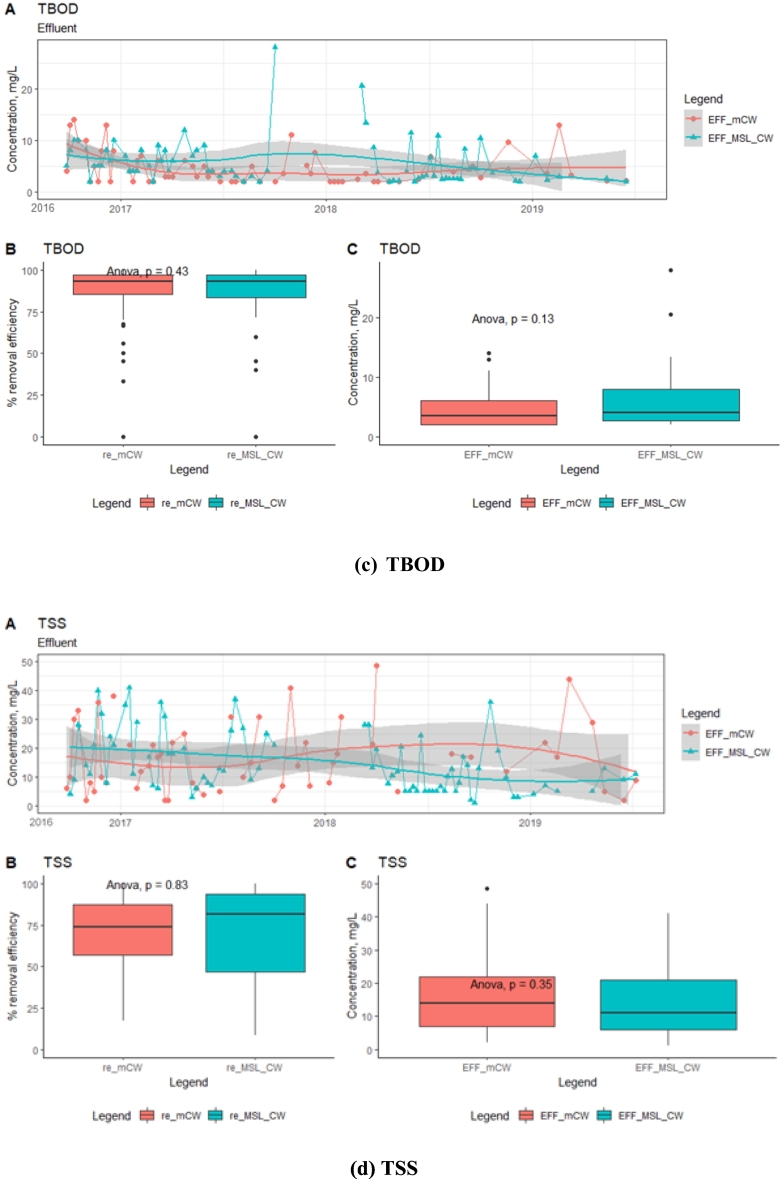
Time-series analysis of effluent concentrations and box plots (removal efficiency (re) and concentration (EFF)): TCOD, SCOD, TBOD, TSS.

The average removal efficiencies of TCOD were found to be 70–72% in both the MSL-CW and mCW units (Fig. 4). Similarly, the removal efficiencies of SCOD and TBOD in the MSL-CW were not significantly different (*p* < 0.05) when compared with the mCW unit, which were 63–68% and 78–82%, respectively. During the 4-year operation, there was not much variation in the effluent concentrations of organic matters of the MSL-CW and mCW, with the average TCOD concentrations of 35 ± 18 and 34 ± 15 mg/L, respectively, followed by SCOD (24 ± 14 and 26 ± 12 mg/L, respectively) and TBOD (5 ± 3 and 6 ± 4 mg/L, respectively). The concentrations of TCOD in the effluent of the MSL-CW and mCW units over the 4-year operation were lower than the global standard (ISO30500, Category A and B) of ≤50 mg/L. During the operation of the systems, the average TSS concentrations in the effluent of the MSL-CW and mCW was 17 ± 15 and 42 ± 85 mg/L, respectively. Possibly due to the washout of solids particles from the beds, the variation of TSS concentrations in the effluent of the mCW was found to be relatively high in comparison with the MSL-CW (Fig. 4d). Therefore, these systems are suitable for TSS reduction and the highest TSS removal efficiency was achieved in the MSL-CW. These effluent concentrations of the MSL-CW were lower than the discharge limit of the ISO30500, Category B for TSS of ≤30 mg/L.

**Fig. 5:**
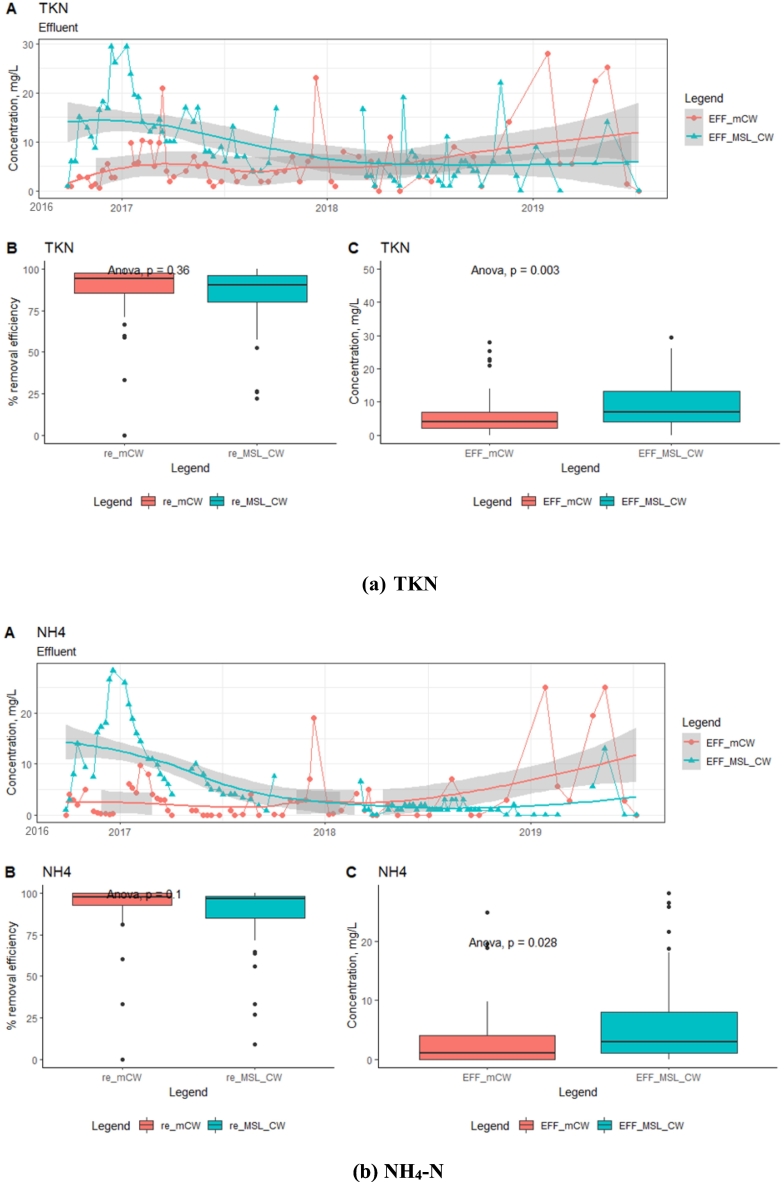

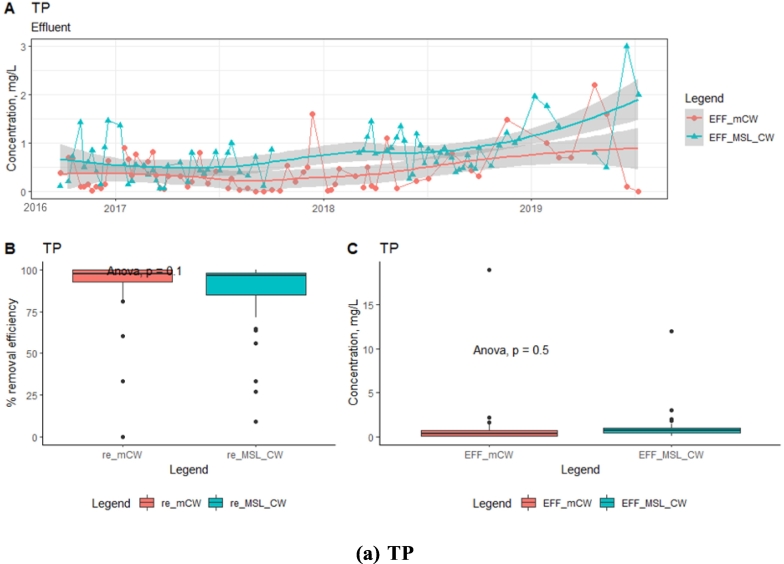
Time-series analysis of effluent concentrations and box plots (removal efficiency (re) and concentration (EFF)): TKN, NH_4_-N and TP.

Statistical analysis for variation in the effluent concentrations of TKN and NH_4_-N revealed that there was a significant difference (*p* < 0.05 concentrations) between the two units. The TKN and NH_4_-N concentrations in the mCW effluent showed a decreasing trend till 2017 and increased at the later years of operation 2017–2018. While, the MSL-CW contained the zeolite in the PLs layer could contribute to NH_4_^+^-N exchange/adsorb in the first year, but as the media become saturated with the short time and the effluent concentration increased from mid-2016 to 2017. A decreasing trend of the TKN and NH_4_-N concentrations in the MSL-CW effluent was significantly observed after 2017. The decreasing trend of the TKN and NH_4_-N concentrations in the MSL-CW was probably due to the simultaneous existence of nitrification-denitrifications reactions with various microbial abundances inside the SMB and PL, which facilitated the conversion of TKN and NH_4_-N. This suggests that the MSL-CW could achieve better TKN and NH_4_-N removal efficiencies in the long run. The zeolite material in the MSL-CW system adsorbs the NH_4_^+^-N ions in the wastewater through ion exchange mechanism and serves as carrier for nitrifying bacteria to convert the intermediate nitrogen (Nitrate nitrogen, NO_3_-N), which can translocate to the SMB, and promote the denitrification under anaerobic conditions (Chen et al., 2009). The effluent concentrations of TP were found in the same magnitude for both units, being about 1 ± 2 mg/L. The high proportions of iron in the laterite soil and porous material contained in the MSL-CW and mCW beds and porous material are hypothesized to be responsible for P adsorption and removal from the influent wastewater (Fig. 5).

**Fig. 6:**
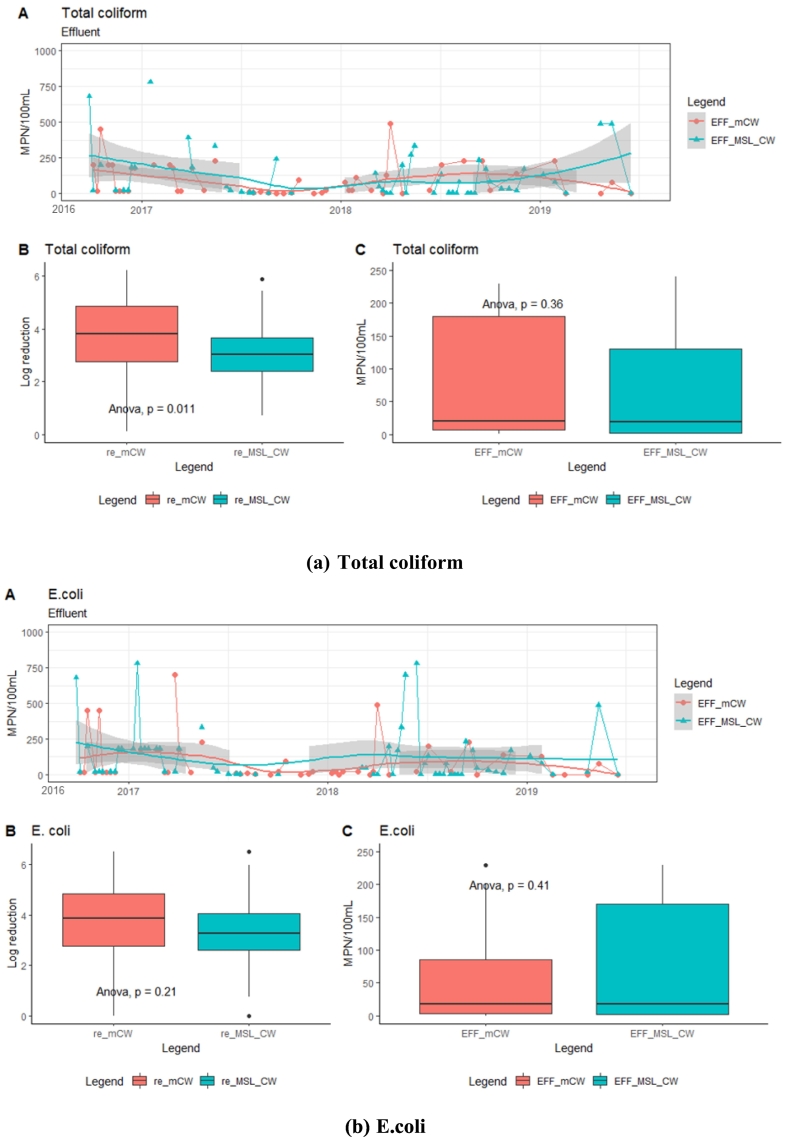
Time-series analysis of effluent concentrations and box plots (log reduction and effluent concentration (EFF)): Total coliform and E.coli.

Although the initial numbers of total coliform and E.coli counts were 6.6 (+1.8) x 10^5^ and 8.0 (2.5) x 10^5^ MPN/100 mL, respectively, they were reduced to be lower than 1000 MPN/100 mL in the MSL-CW and mCW effluents (Fig. 6). The reduction of E.coli depended on the porosity of soil by adhesion in soil media layer (Kadam et al., 2008). The relatively high pathogen removal in the MSL-CW and mCW units suggests a potential to reuse their treated effluent in the cultivation of perennial crops or discharge to receiving water (WHO, 1989). During the 4- year operation, average E.coli concentrations in the effluents in both units were slightly higher than the ISO standard of <10 MPN/100 mL, even without any disinfection unit. The treated effluent from the MSL-CW and mCW can be used for different purposes, such as garden watering, lawn irrigation and toilet flushing and utilizes an on-site resource which would otherwise be wasted.

### 3.2 Annual performance and effects of seasonal variations

**Fig. 7:**
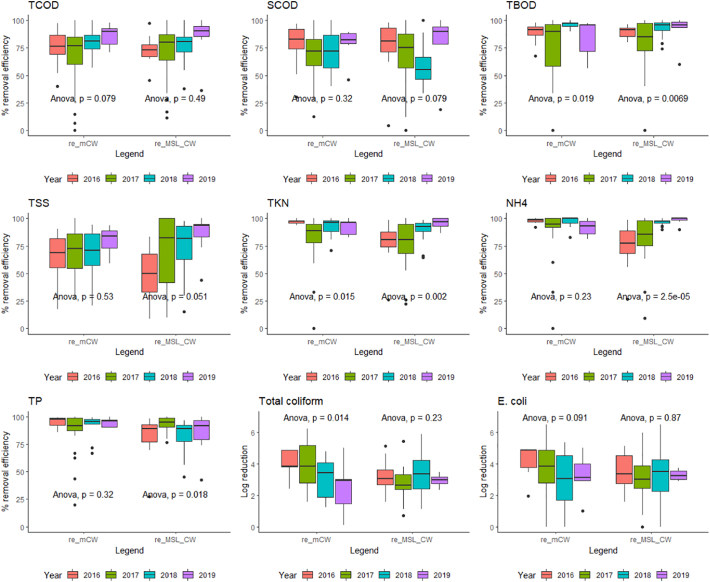
Box plots of yearly removal efficiency (re): mCW and MSL.

The average removal efficiencies of TCOD and TSS of the mCW and MSL-CW units during the operation period of 2016–2019 were found in the range of 74–85% and 72–84%, respectively (Fig. 7). There was no significant difference (*p* > 0.05) among the data of each year. Due to the availability of readily degradable organics, the removal efficiencies of TBOD were found to have the same trend between the MSL-CW and mCW units. Increasing trends of the SCOD, TKN and NH_4_-N removals in the MSL-CW unit were observed in the long-term operation with the *p* < 0.05. Possibly due to the high porosity and nutrients/carbon-rich media of the SMBs, the MSL-CW unit could support active growth of microorganisms and consequently enhancing the removal of organic matters and nitrogen. Over a long term operation, the designed multi solid layers systems could likely provide stability to the base media in subsurface infrastructure and minimize the decrease in their drainage capacity. In this study, the MSL-CW and mCW could thus dramatically reduce the clogging problem or the accumulation of fine micron-sized particles. The clogging issues or the solids accumulation in long-term operation (more than 10 years) of the MSL-CW and mCW would be recommended for further investigation. Several studies indicated that the actual operations of the conventional constructed wetland (CW) longevity below 2 years, and thus some degree of clogging in the CW medium is inevitable (Grismer et al., 2003; de la Varga et al., 2013).

**Fig. 8:**
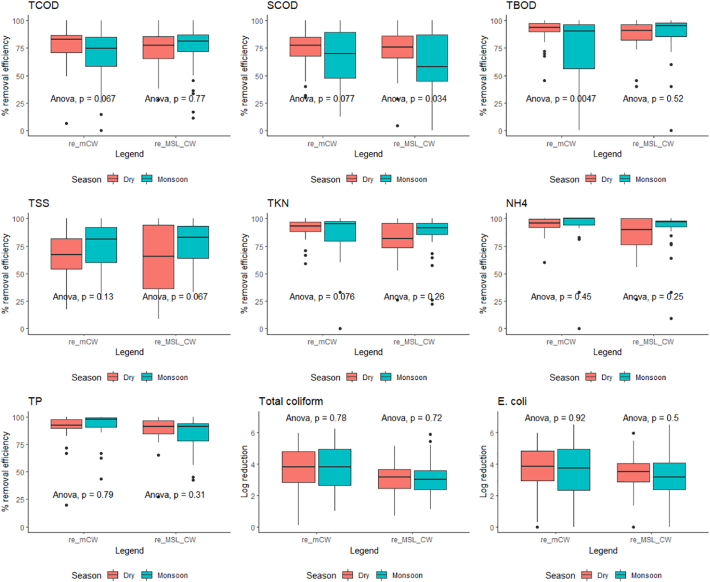
Effect of seasonal variations on the treatment performance: mCW and MSL (removal efficiency, re)

With respect to tropical seasons in Thailand i.e. dry (November to April) and monsoon (May to October), the average removal efficiencies of the mCW unit during the dry season was relatively higher (*p* < 0.05) for TCOD, SCOD, TBOD and TKN, while the average removal efficiencies of NH_4_-N, TP, total coliform and E.coli were no significantly different from those obtained during monsoon months (*p* > 0.05) (Fig. 8). Probably due to large temperature fluctuations (5–10 °C) between wet and dry periods with monsoonal rains which could influence the processes of microbial transformation, the removal efficiencies of the mCW unit were significantly lower for TCOD (18%), SCOD (15%), TBOD (19%) and TKN (12%) than those obtained during the dry season. On the contrary, it seems no seasonal effect on removal efficiencies of the MSL-CW unit (p > 0.05) in TCOD, TBOD, TSS, TKN, NH_4_-N, TP, total coliform and E.coli removal efficiencies. The relatively stable performance of the MSL-CW unit was probably due to the SMBs and PLs inside the unit could enhance microbial tolerance to the seasonal variations.

### 3.3 Effects of plant harvesting on treatment performance

**Fig. 9:**
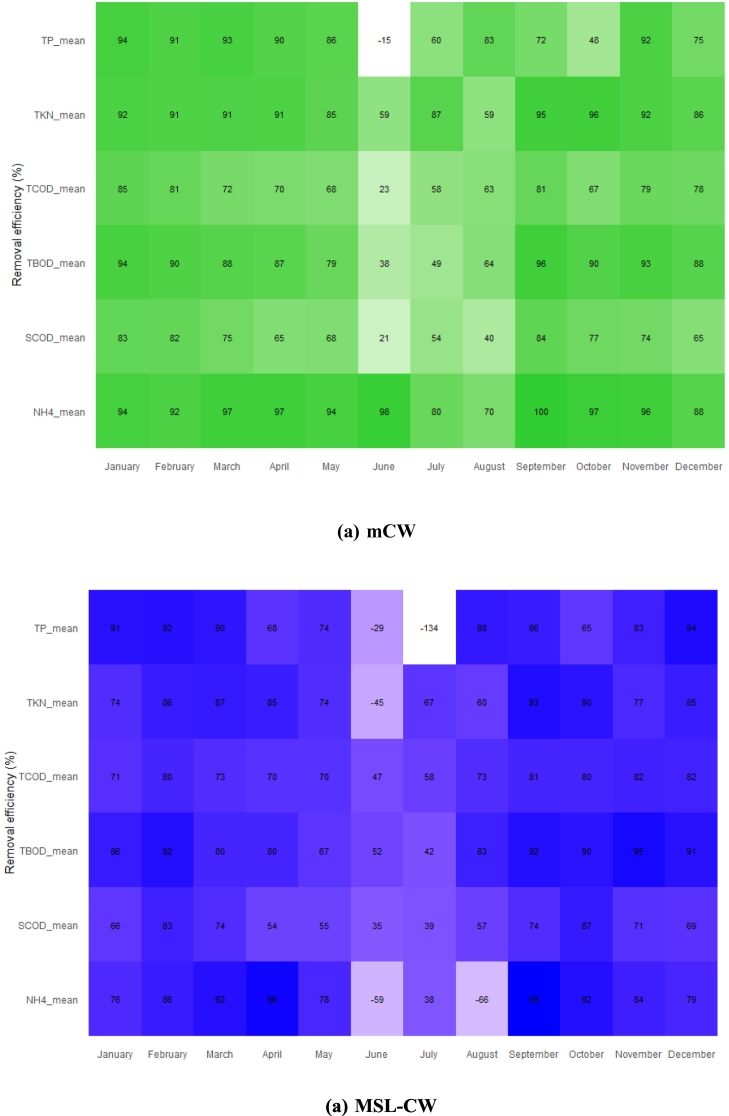
Heat map of average (mean) monthly treatment performance: (a) mCW and (b) MSL-CW.

During the operation period, the plant shoots were harvested in January (1st harvest), May (2nd harvest) and September (3rd harvest) annually, and the monthly average of the removal efficiencies of MSL-CW and mCW units are shown in Fig. 9. The removal efficiencies of TCOD, SCOD, TBOD, TKN, NH_4_-N and TP of the 1st and 3rd harvests were not significantly different between the 1-month before and 2-months after harvesting in both units. It might be hypothesized that the growing season of these plants could not contribute to the greater adsorption of pollutants after harvesting. In contrast, after the 2nd harvest, the lower removal efficiencies in TCOD, SCOD, TBOD, TKN, NH_4_-N and TP were found after 2–3 months period from June to August in both units. These results could possibly be affected by heavy precipitation in monsoon season causing high flow rate of water through the MSL-CW and mCW beds and flushing out the pollutants.

For the prototype of a single family, the MSL-CW or mCW system thus provide a cost-effective means to treat water pollution *locally* and the total investment was about US$ 1000-1500. In situ locally available medias (zeolite, laterite soil, gravel and other) were used for the construction of the MSL-CW or mCW system which could cost more than 80% of the total investment. However, it should be noted that the filed test (or prototype unit) should thus be an integral part of the design process and not be used only to consider as a final product and the commercialized product.

## 4 Conclusions

The MSL-CW and mCW units are considered as novel constructed wetlands for efficiently treating effluent of onsite sanitation systems including solar septic tank. A long-term operation of pilot-scale MSL-CW and mCW units were undertaken to demonstrate the applicability of these novel constructed wetlands. The monitoring results indicated that both units are very effective in removing organic matters, solid, nutrients and pathogens and able to meet the global standard of ISO30500 and WHO guidelines for wastewater reuse. Based on the results of this study, specific conclusions can be made as follows:

– TCOD, SCOD and TBOD removals of the MSL-CW and mCW units were not significantly different (*p* < 0.05), which were 70–72%, 63–68% and 78–82%, respectively, while TSS, TKN, NH_4_-N and TP removals were found greater than 70%.
– Total coliform and E.coli counts in the effluent of both MSL-CW and mCW units were apparently reduced at the concentration lower than 10^2^ MPN/100 mL without disinfection unit.
– Increasing trends of SCOD, TKN and NH_4_-N removals in the MSL-CW unit were observed since 2016 to present with the *p* < 0.05.
– Removal efficiencies of the mCW unit during the monsoon season were significantly lower for TCOD, SCOD, TBOD and TKN than during dry season; while removal efficiencies of the MSL-CW unit were relatively stable during both the dry and monsoon seasons.
– Removal efficiencies in terms of TCOD, SCOD, TBOD, TKN, NH_4_-N and TP during the 1st and 3rd harvests were not significantly different between the 1-month before and 2-months after harvesting in both units.
– A novel designed media in the vertical-flow CW system could apparently limit clogging problem during 4-year operation.

## Declaration of competing interest

We have no conflict of interest to declare.
